# A Novel High Sensitivity Sensor for Remote Field Eddy Current Non-Destructive Testing Based on Orthogonal Magnetic Field

**DOI:** 10.3390/s141224098

**Published:** 2014-12-12

**Authors:** Xiaojie Xu, Ming Liu, Zhanbin Zhang, Yueling Jia

**Affiliations:** 1 School of Information and Navigation, Air Force Engineering University, Xi'an 710077, China; E-Mails: lming0601@163.com (M.L.); zzbin@163.com (Z.Z.); 2 School of Equipment Management and Safety Engineering, Air Force Engineering University, Xi'an 710077, China; E-Mail: jyl1974@126.com

**Keywords:** non-destructive testing, remote field eddy current, sensor, orthogonal magnetic field, finite element simulation

## Abstract

Remote field eddy current is an effective non-destructive testing method for ferromagnetic tubular structures. In view of conventional sensors' disadvantages such as low signal-to-noise ratio and poor sensitivity to axial cracks, a novel high sensitivity sensor based on orthogonal magnetic field excitation is proposed. Firstly, through a three-dimensional finite element simulation, the remote field effect under orthogonal magnetic field excitation is determined, and an appropriate configuration which can generate an orthogonal magnetic field for a tubular structure is developed. Secondly, optimized selection of key parameters such as frequency, exciting currents and shielding modes is analyzed in detail, and different types of pick-up coils, including a new self-differential mode pick-up coil, are designed and analyzed. Lastly, the proposed sensor is verified experimentally by various types of defects manufactured on a section of a ferromagnetic tube. Experimental results show that the proposed novel sensor can largely improve the sensitivity of defect detection, especially for axial crack whose depth is less than 40% wall thickness, which are very difficult to detect and identify by conventional sensors. Another noteworthy advantage of the proposed sensor is that it has almost equal sensitivity to various types of defects, when a self-differential mode pick-up coil is adopted.

## 1. Introduction

**Figure 1.**
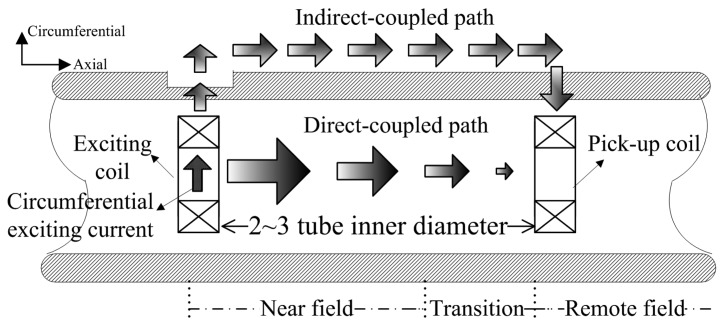
Principle of remote field eddy current testing.

Eddy current testing is one of the most extensively used non-destructive testing (NDT) methods for conductive materials [1]. Remote field eddy current (RFEC) is a type of eddy current NDT, and has drawn more and more attention in the nondestructive testing of ferromagnetic tubular structures. RFEC has remarkable advantages such as almost equal sensitivity to inner and outer defects, easy defect characterization and insensitivity to lift-off or wobble [2]. Remote field eddy current testing mainly depends on indirect-coupled electromagnetic energy, which passes through a pipe wall twice, as shown in Figure 1.

Detection sensitivity of RFEC testing, defined as the signal variation caused by a defect relative to a defect free sample, largely depends on the performance of its sensor. A conventional sensor uses a solenoid as an exciting coil and another co-axial solenoid 2 ∼ 3 tube inner diameters away as a pick-up coil. When the conventional sensor is used for ferromagnetic structures testing, it has disadvantages such as low signal-to-noise ratio (SNR) and poor sensitivity to axial cracks. Low SNR can be overcome by advanced signal detection and processing technology, for example, by using a lock-in amplifier to improve the weak signal detection ability and a homomorphic filter to eliminate the influence of nonlinear noise such as a magnetic perturbation [3]. However, its poor sensitivity to axial cracks is not easy to solve, because a conventional sensor only provides an axial magnetic field, which is more sensitive to a circumferential crack than an axial cracks. Some people try to solve it by using an appropriate pick-up coil to detect the weak electromagnetic field perturbation, such as differential modes and radial field pick-up coils [4]. On the other hand, some try to change the direction of the excitation to generate a specified electromagnetic field, for example, charging electricity directly on the tube wall to generate circumferential magnetic field [5], using an induction motor as excitation to detect stress corrosion cracks [6] and an inclined exciting coil to detect cracks in different directions [7]. Until now, how to design a sensor with appropriate exciting coils and pick-up coils is still critical to the sensitivity improvement of remote field eddy current testing. This article aims to demonstrate a remote field effect under orthogonal magnetic field excitation and design a novel sensor based on it.

## 2. Orthogonal Magnetic Field for Tubular Structures

### 2.1. Principle of the Orthogonal Magnetic Field

In conventional eddy current testing, orthogonal magnetic field excitation is proposed to inspect tabular structures. Two windings are located on the perpendicular plan of a rectangular frame, and the two windings are excited respectively by a sinusoid with the same frequency but quadrature lagging, and thus the magnetic fields generated by the two windings are oriented at 90° to each other—called orthogonal magnetic field excitation [8].

The magnetic flux density generated by the two windings can be described as follows:

(1) $$B_{x}=A_{1}\sin\left(\omega t+\varphi_{0}\right)$$

(2) $$B_{y}=A_{2}\cos\left(\omega t+\varphi_{0}\right)$$

where A_1_ and A_2_ are the amplitude of magnetic flux density, ω is the exciting frequency and φ_0_ is the initial phase. B_x_ and B_y_ meet the elliptic equation:

(3) $$\frac{{B_{x}}^{2}}{{A_{1}}^{2}}+\frac{{B_{y}}^{2}}{{A_{2}}^{2}}=\sin{\left(\omega t+\varphi_{0}\right)}^{2}+\cos{\left(\omega t+\varphi_{0}\right)}^{2}=1$$

When A_1_ = A_2_ = A, Equation (3) can be simplified to a standard circular equation, and the combined magnetic flux density can be calculated by:

(4) $$AMP_{B}=\sqrt{A^{2}\sin^{2}\left(\omega t+\varphi_{0}\right)+A^{2}\cos^{2}\left(\omega t+\varphi_{0}\right)}=\;\text{A}$$

(5) $$\begin{array}{l} {\text{Phase}}_{B}=\mathrm{atan}\left(B_{x}/B_{y}\right)=\mathrm{atan}\left(\sin\left(\omega t+\varphi_{0}\right)/\cos(\omega t+\varphi_{0})\right)\\ =\mathrm{atan}\left(\tan\left(\omega t+\varphi \right)\right)=\left(\omega t+\varphi_{0}\right) \end{array}$$

From Equations (4) and (5), the strength of the combined magnetic field is constant and its direction rotates with time periodically. According to the Maxwell equation *J̇* = ∇ × *Ḣ*, the eddy current induced in the specimen follows the same rule. Owing to its rotating characteristics, the above-mentioned orthogonal magnetic field excitation is proved to be sensitive enough to cracks in different directions.

The abovementioned coil mainly relies on a rectangular frame, and is not suitable for tubular structures because of the big air gap between the windings and tube wall. In [9–11], the authors propose a new exciting coil with three orthogonal coils making a 2π/3 angle between and claim its remote field effect. In [5], the authors researched the remote field effect under circumferential magnetic field excitation by charging electricity directly on the tube wall, and claim an improvement of detection sensitivity to axial cracks. Considering the advantages of the abovementioned methods, the orthogonal pattern combined with axial magnetic field and circumferential magnetic field should be appropriate for the RFEC testing of tubular structures, with minimum modification of conventional sensors to get high sensitivity to different types of defects.

### 2.2. Finite Element Simulation

Three-dimensional finite element method (FEM) is an effective simulation tool for eddy current NDT [12–16], and is adopted to verify the applicability of the proposed orthogonal magnetic field excitation pattern combined with axial magnetic field and circumferential magnetic field. Table 1 shows the simulation conditions.

**Figure 2.**
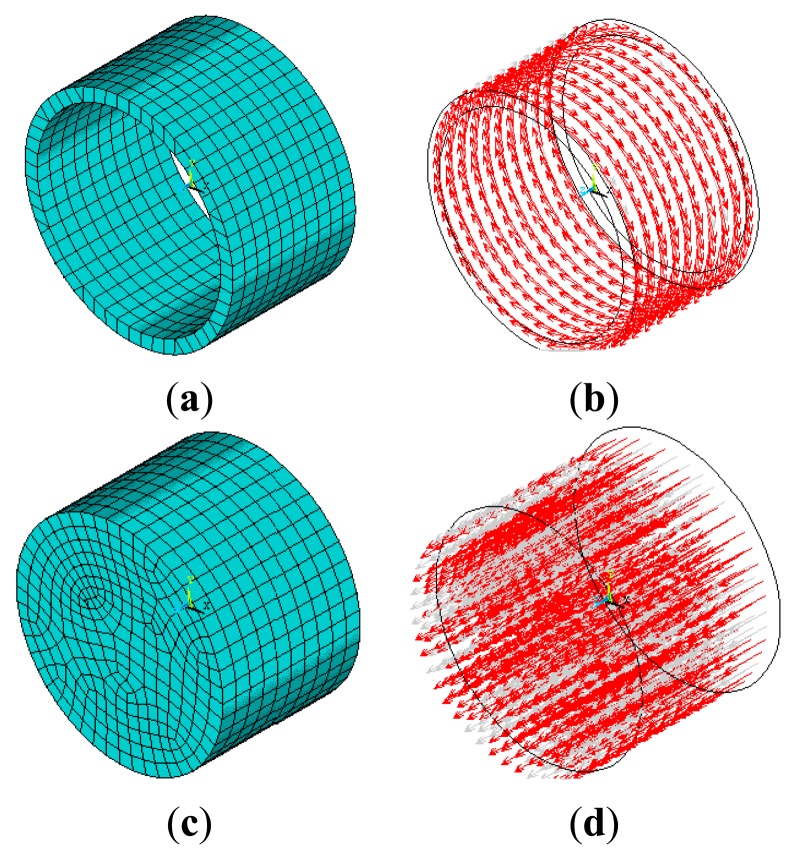
(**a**) Solenoid exciting coil; (**b**) Circumferential current density; (**c**) Metal cylinder; (**d**) Axial current density.

Figure 2 shows the simulation models of the exciting coil. Figure 2a shows finite element mesh of the conventional solenoid exciting coil, while Figure 2b shows the circumferential current density applied on the coil, which is used to generate axial magnetic field. Figure 2c shows the finite element mesh of a metal cylinder located inside the conventional solenoid, while Figure 2d shows the axial current density applied on the metal cylinder, which is used to generate a circumferential magnetic field.

**Figure 3.**
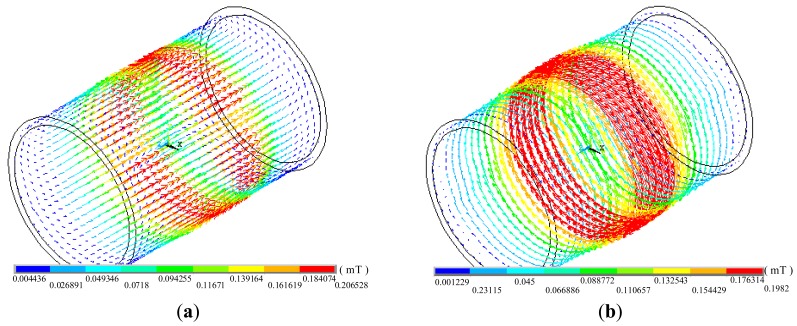
(**a**) Real part of magnetic flux density; (**b**) Imaginary part of magnetic flux density.

Figure 3 shows the real part and imaginary part of spatial magnetic flux density, and the two parts correspond to axial magnetic field excitation and circumferential magnetic field excitation. The axial magnetic flux density (near field is 0.206528 mT, and remote field is 0.094255 mT) and circumferential magnetic flux density (near field is 0.1982 mT, and remote field is 0.088772 mT) are almost the same, indicating that the simulation models and parameters are suitable for generating almost equal axial magnetic field and circumferential magnetic field.

**Figure 4.**
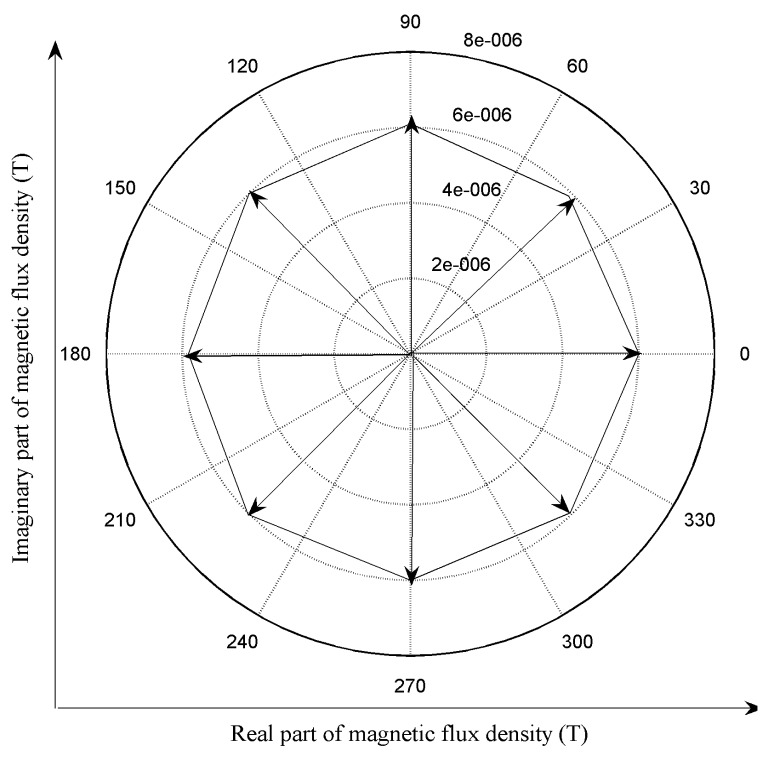
Changes of magnetic flux density direction.

Figure 4 shows periodic changes of magnetic flux density direction at a fixed point in the remote field zone. The variation rule is very similar to the orthogonal magnetic field under a rectangular frame, which means that the remote field effect is still valid under a combination of axial magnetic field excitation and circumferential magnetic field excitation.

### 2.3. Comparison of Conventional Axial Field Excitation and Orthogonal Field Excitation

**Figure 5.**
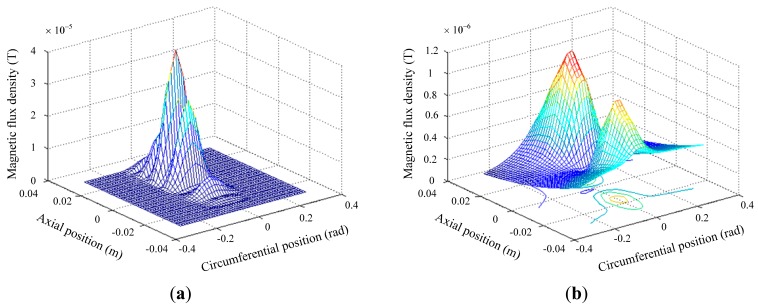
Perturbation field caused by (**a**) Circumferential crack; (**b**) Axial crack.

To facilitate the analysis, a perturbation field computed by the “anomalous source” method is used, which means removing the defect free background field from the computed field when there is a defect [17]. Firstly, the crack detection ability of the conventional exciting coil is simulated and the radial perturbation fields around the axial and circumferential crack are shown in Figure 5. The perturbation caused by the circumferential crack (3.0 × 10^−5^ T) is even thirty three times higher than with the axial crack (0.9 × 10^−6^ T). The simulation result can be used to interpret the poor detection sensitivity to axial cracks.

**Figure 6.**
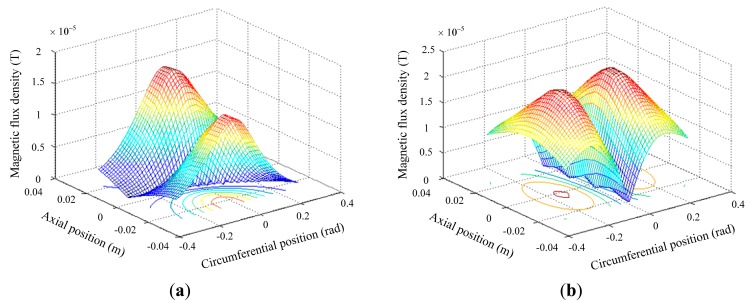
Perturbation field caused by (**a**) Circumferential crack; (**b**) Axial crack.

Secondly, the crack detection ability of the proposed orthogonal magnetic field excitation is simulated and radial perturbation field around the axial and circumferential crack are shown in Figure 6. The perturbation caused by the circumferential crack (1.3 × 10^−5^ T) is almost the same as that by the axial crack (1.38 × 10^−5^ T), which means the proposed exciting coil can achieve high sensitivity to cracks in different directions.

## 3. Novel Sensor Design

**Figure 7.**
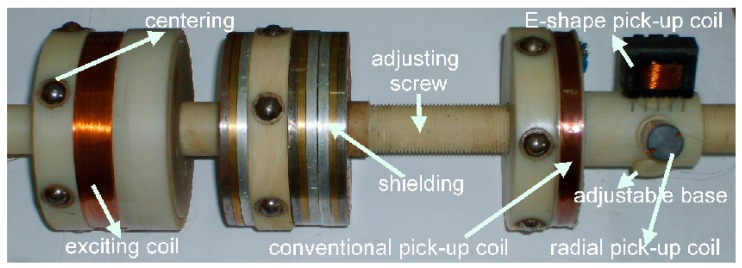
Composition of proposed novel sensor.

Figure 7 shows the composition of the proposed novel sensor. The sensor consists of an exciting coil, a centering device, and different types of pick-up coils, an adjustable base, an adjusting screw and a shielding facility. The exciting coil is used to obtain the orthogonal magnetic field, and the centering device to control the sensor's wobble or off-center movement. The pick-up coils of different types with an adjustable base are used to detect different components of the magnetic field perturbation. The adjusting screw is used to set a proper distance between the exciting coil and pick-up coil. The shielding facility is used to prevent the direct-coupled magnetic field from entering the pick-up coil, and thus reduce the sensor's length.

### 3.1. Exciting Coil Design

**Figure 8.**
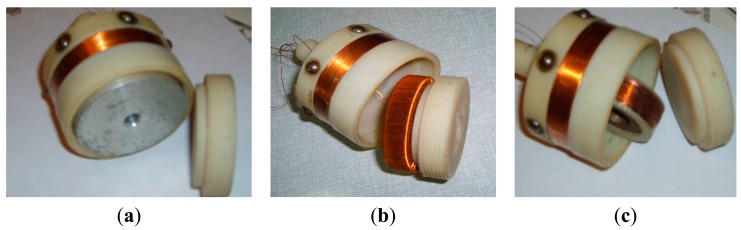
(**a**) Configuration 1; (**b**) Configuration 2; (**c**) Configuration 3.

Once another coil which can obtain circumferential magnetic field is designed and combined with the existing solenoid coil, the orthogonal magnetic field will be obtained. Figure 8 shows the interior of the exciting coil, and three possible configurations which can generate the orthogonal magnetic field for tubular structures are shown in Figure 8a–c, respectively.

Configuration 1 adds a coaxial cylindrical conductor inside a conventional solenoid, as shown in Figure 8a. When an AC current is applied between two ends of the cylindrical conductor, due to the skin effect, the current will be collected on the surface [18,19], and then the circumferential magnetic field will be obtained.

Configuration 2 adds a spiral coil inside a conventional solenoid, as shown in Figure 8b. According to the right-hand rule, a circumferential magnetic field will be obtained. Although the circumferential magnetic field will be restricted in the magnetic core in ideal conditions in accordance with Ampere's circuit theorem, some circumferential magnetic flux will leak to the surrounding air, as the ferromagnetic tube wall can serve as another magnetic path [20].

Configuration 3 adds a solenoid which is perpendicular to a conventional solenoid, as shown in Figure 8c. According to the right-hand rule, a radial magnetic field will be obtained. Some of the radial magnetic field will propagate along the circumferential surface of the tube, as the ferromagnetic tube wall can serve as another magnetic path.

**Figure 9.**
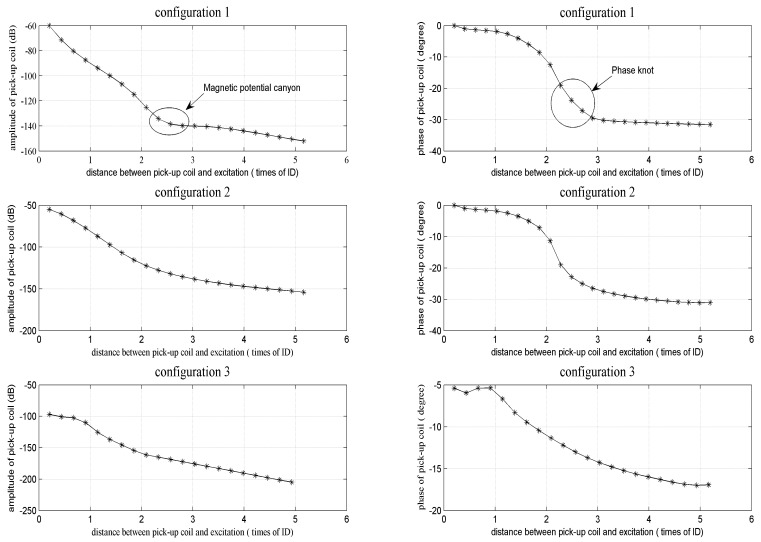
Comparison results of three configurations.

Two important phenomena of remote field eddy current testing: the “magnetic potential canyon” which means the transition zone where fast signal attenuation changes to slow signal attenuation, and “phase knot” which means the phase difference between a near field and a remote field, are used for the comparison of the three configurations mentioned above. Figure 9 shows “pull experiment” results, which are achieved by taking the pick-up coil away from the exciting coil step by step. The “magnetic potential canyon” and “phase knot” phenomena can be observed clearly when the first and second configuration (*i.e.*, Configurations 1 and 2) is used, but the third configuration failed. Finally, the first configuration is considered as the most appropriate one, because the remote field zone is relatively closer to the exciting coil (3 tube inner diameter) than the second (3.8 tube inner diameter), which means a shorter sensor can be designed.

### 3.2. Pick-Up Coil Design

#### 3.2.1. Differential Mode

Because the remote field eddy current is a relatively weak signal system, a differential mode pick-up coil is usually used to improve the detection sensitivity [21,22]. Adopting two rod-shape pick-up coils is the most popular differential mode used for eddy current nondestructive testing. The differential of output signals of two windings can improve the detection sensitivity, however, it is susceptible to wobble or off-center movement. At the same time, it is difficult to ensure uniformity of the two windings. Two U-shape pick-up coils, which use two windings warped on two “face to face” U-shape magnetic cores, can reduce the influence of wobble or off-center movement, for the two windings sharing same magnetic paths. Yet, it is still difficult to deal with the uniformity of the two windings.

**Figure 10.**
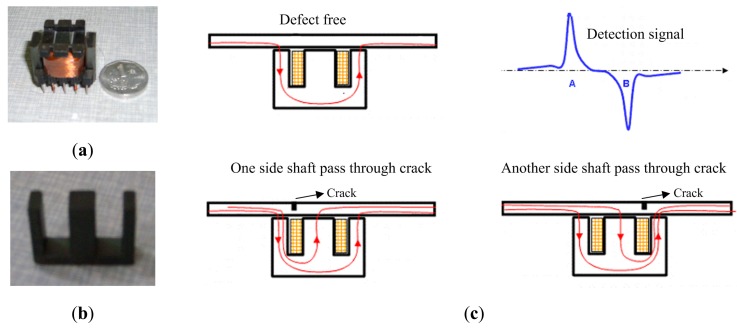
Self-differential mode (**a**) Pick-up coil; (**b**) E-shape magnetic core; (**c**) Principle.

**Figure 11.**
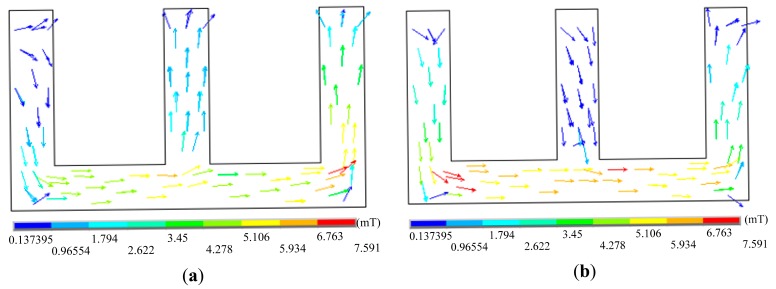
(**a**) One side shaft pass through crack; (**b**) Another side shaft pass through crack.

A pick-up coil with an E-shape magnetic core shown in Figure 10 is a new differential mode, which was first proposed to detect cracks in aircraft multilayer structures [23], and is called “self-differential”. The pick-up coil is wound on the central axis of the E-shape magnetic core, as shown in Figure 10a–c which shows its principle. In the symmetric case which is defect-free, there is no magnetic flux that enters the central axis, because the magnetic flux always follows the path which has minimum reluctance in accordance with magnetic path theory. When one of the side shafts passes through a defect, the symmetry will be broken because of the stronger magnetic field intensity around the defect, and then a part of magnetic flux will enter the central axis from the bottom to the top and is picked up by the winding. When another side shaft passes through a defect, a part of magnetic flux will enter the central axis from the opposite direction. Figure 11 shows the simulation results. Figure 11a displays the magnetic field distribution when one side shaft passes through crack, and the direction of magnetic flux in the central axis is from the bottom to the top. Figure 11b displays the magnetic field distribution when another side shaft passes through a crack, and the direction of the magnetic flux in central axis is from the top to the bottom.

#### 3.2.2. Conventional Pick-Up Coil, Circumferential and Radial Field Pick-Up Coils

**Figure 12.**
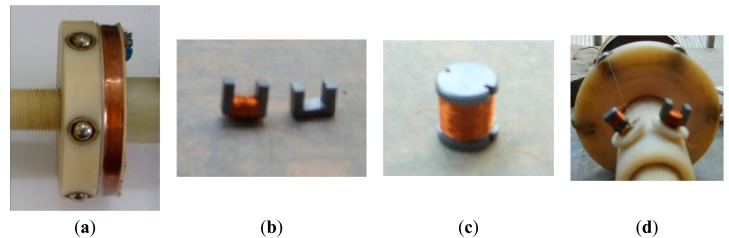
(**a**) Conventional pick-up coil; (**b**) Circumferential field pick-up coil; (**c**) Radial field pick-up coil; (**d**) Placement.

Conventional pick-up coils and local pick-up coils which are sensitive to the circumferential field and radial field are also designed. Figure 12a shows the conventional pick-up coil, which is a co-axial solenoid. Figure 12b shows the circumferential field pick-up coil based on a U-shape magnetic core. Figure 12c shows the radial field pick-up coil based on a rod-shape magnetic core. Figure 12d shows their placement.

### 3.3. Experimental System Design

**Figure 13.**
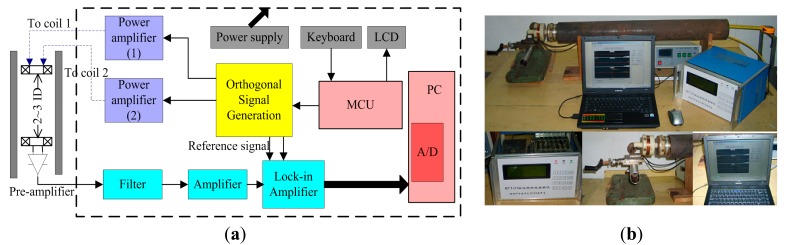
(**a**) Schematic diagram of experimental system; (**b**) Photograph of experimental system.

Figure 13 shows the schematic diagram and photography of the experimental system, which consists of orthogonal signal generation units, power amplifier units, power supply units, display units and signal acquisition and processing units.

### 3.4. Key Parameters Selection

#### 3.4.1. Frequency

**Figure 14.**
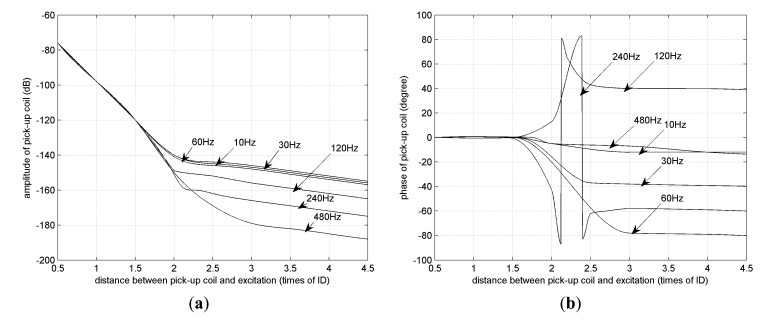
Characteristic curve under different frequency. (**a**) Amplitude; (**b**) Phase.

Figure 14 shows RFEC characteristic curves under different frequency conditions (10, 30, 60, 120, 240 and 480 Hz). When the frequency increases, the signal strength decreases rapidly, as shown in Figure 14a, because high frequency electromagnetic waves are attenuated dramatically when they pass through the tube wall twice. This is the reason why RFEC testing of ferromagnetic tubes is always done at frequencies below 60 Hz. Figure 14b shows the phase difference between a remote field zone and a near zone. Except for the lowest frequency (10 Hz) and highest frequency (480 Hz), the “phase knot” effect can be clearly observed, and the maximum appears at 60 Hz (about 60° phase difference), indicating that a frequency between 30 and 240 Hz can be appropriate. Finally, 60 Hz is selected as the exciting frequency, for proper signal strength and maximum phase difference which will be helpful for crack identification.

#### 3.4.2. Exciting Current

**Figure 15.**
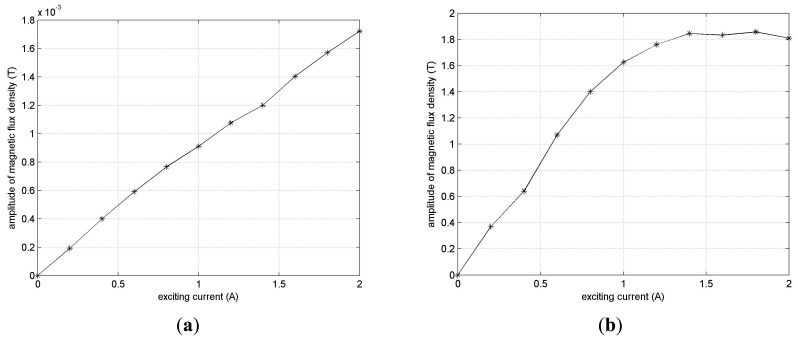
Influence of exciting currents. (**a**) Simulation; (**b**) Experiment.

The remote field eddy current is a low energy transmission system, and the amplitude of a pick-up coil in the remote field zone usually has a few millivolts. An exciting current properly selected can increase the signal strength. Figure 15 shows the influence of different exciting currents.

The relationship between signal strength and exciting currents remains nearly linear in FEM simulation, as shown in Figure 15a. However in the experimental test, a nonlinear relationship appears when the exciting current increases to 1.4 A or more. In some cases, the signal strength even decreases with the increase of the exciting current, as shown in Figure 15b. This is perhaps because the ferromagnetic tube will enter the so-called Rayleigh region when the exciting current is high enough, and then the tube's permeability will increase. In view of RFEC testing's dependence on energy passing twice through the tube wall, the increasing permeability will reduce the energy coupled to the pick-up coil, and then decrease signal strength. Finally, the exciting current of the solenoid and cylindrical conductor is set at 1.1 A and 1.7 A respectively to ensure similar strength of the axial and circumferential field in a remote field zone.

#### 3.4.3. Shielding Facility

**Figure 16.**
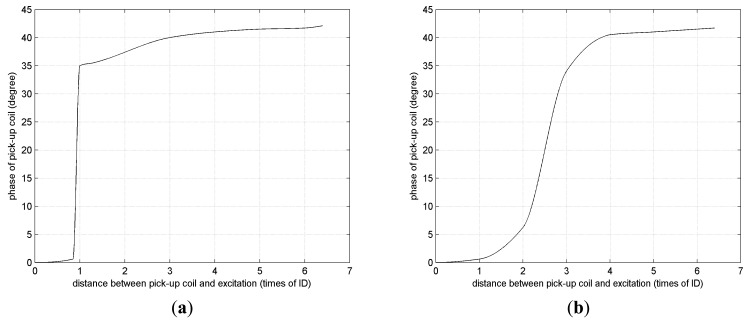
RFEC Characteristic curves. (**a**) With shielding facility; (**b**) without shielding facility.

In the remote field eddy current testing system, a pick-up coil should be located about 2 ∼ 3 times the inner diameter away from an exciting coil. Taking into account the influence of wobble or off-center movement, the distance between pick-up coil and exciting coil always be 4 ∼ 5 times the inner diameter. The most effective method to reduce sensor length is to use a shielding facility to prevent the direct-coupled field from entering the pick-up coil. A combination of steel, copper and aluminum shown in Figure 7 is feasible and effective. Figure 16 shows the comparison of RFEC characteristic curves with and without a shielding facility. The remote field zone begins from about 1.1 times of inner diameter by using a shielding facility, as shown in Figure 16a. Taking into account the influence of wobble or off-center movement, the pick-up coil is located 1.7 times of inner diameter away from the exciting coil.

## 4. Experimental Verification

A ferromagnetic tube whose inner diameter is 70 mm, outer diameter 82 mm and wall thickness 6 mm is used as a test specimen. Three types of defects are manufactured on the tube, as shown in Table 2. The above-mentioned self-differential mode pick-up coil, conventional pick-up coil, circumferential field pick-up coil and radial field pick-up coil are used to verify the detection sensitivity of orthogonal magnetic field excitation.

### 4.1. Self-Differential Mode Pick-Up Coil

**Figure 17.**
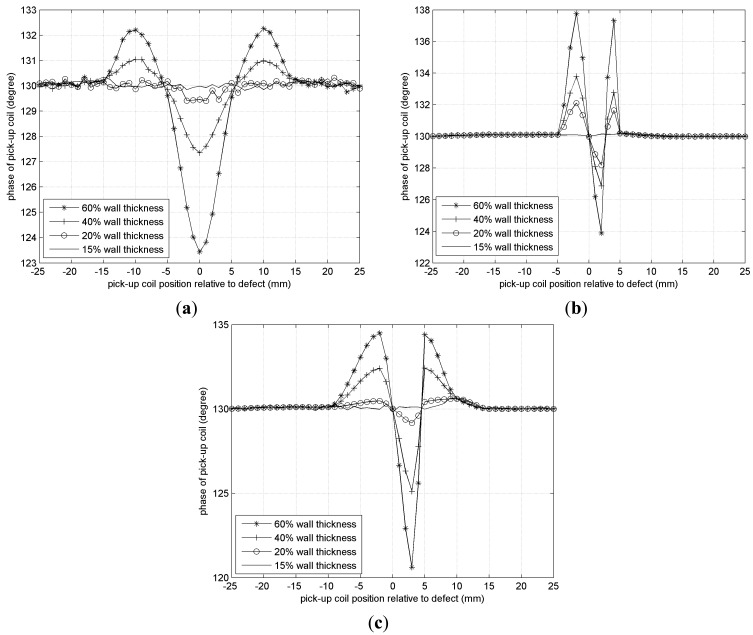
Detection results. (**a**) Axial crack; (**b**) Circumferential crack; (**c**) Circular defect.

Figure 17 shows detection results when a self-differential mode pick-up coil based on an E-shape magnetic core is adopted. Although all of the defects whose depth are 15% wall thickness fail to be detected, the 20% wall thickness axial crack which cannot be detected by a conventional sensor can be detected and identified. At the same time, its detection sensitivity to different defects remains almost the same. For example, detection sensitivity to 60% wall thickness axial crack, circumferential crack and circular defect are 6.7° phase difference, 6.2° phase difference, and 9.6° phase difference respectively, which can be seen from the peak change of Figure 17a–c, respectively.

### 4.2. Circumferential Field Pick-Up Coil

**Figure 18.**
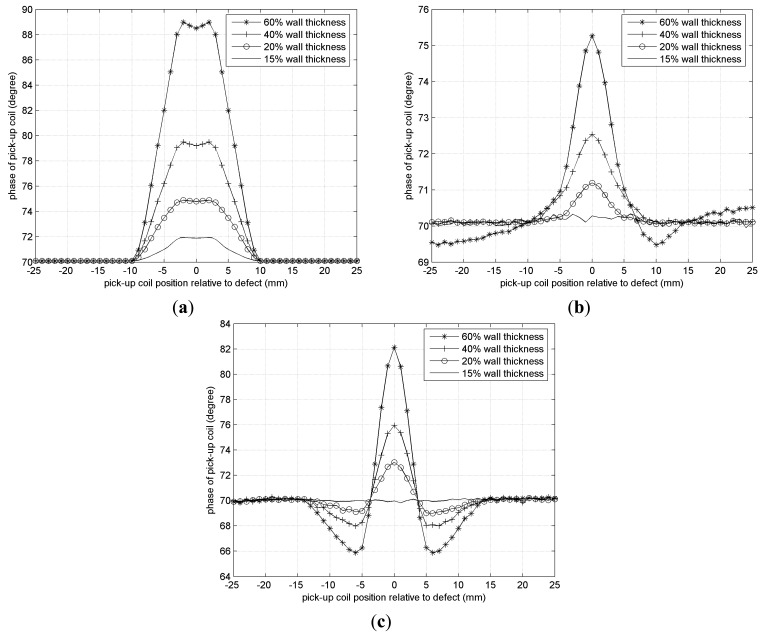
Detection results. (**a**) Axial crack; (**b**) Circumferential crack; (**c**) Circular defect.

Figure 18 shows detection results when a circumferential field pick-up coil based on a U-shape magnetic core is adopted. It is worth noting that a 15% wall thickness axial crack can be detected and identified clearly, but a 15% wall thickness circumferential crack and a circular defect fail to be detected. It means that this type of pick-up coil has enough sensitivity to detect axial cracks, but its detection sensitivity to circumferential cracks (5.1° phase difference) is relatively lower than to axial cracks (18.6° phase difference), which can be seen from the peak change of Figure 18.

### 4.3. Radial Field Pick-Up Coil and Conventional Pick-Up Coil

**Figure 19.**
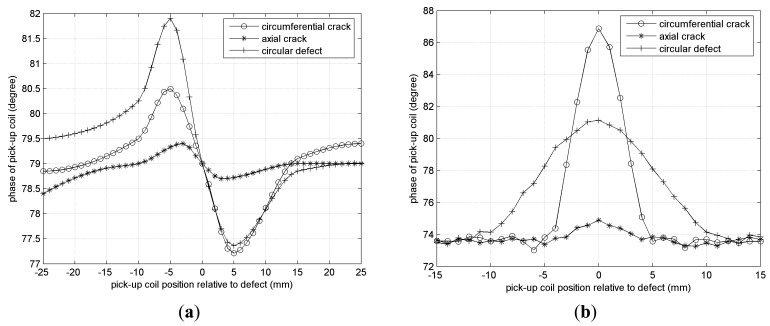
Detection results. (**a**) Radial field pick-up coil; (**b**) Conventional pick-up coil.

When a radial field pick-up coil based on a rod magnetic core is adopted, all of the 15% wall thickness defects fail to be detected. Figure 19a shows the comparison of detection results for 20% wall thickness defects only. The detection results are similar to those of the self-differential mode pick-up coil, but the sensitivity is relatively lower, for instance, only 0.5° phase difference for the 20% wall thickness axial crack. A conventional pick-up coil using a co-axial solenoid can only detect axial cracks whose width is 0.5 mm and depth more than 50% wall thickness. Figure 19b shows the detection results of a 50% wall thickness axial crack, circumferential crack and circular defect by a conventional pick-up coil. The detection sensitivity to circumferential cracks is much higher than that to axial cracks, and it is not easy to identify the axial crack from the background signal because the phase difference only is 0.3°.

## 5. Conclusions

A conventional RFEC sensor has poor sensitivity to axial cracks, because it only uses axial magnetic field excitation. With orthogonal magnetic field excitation, its sensitivity to various types of defects can be improved effectively, especially to axial cracks. Adding a coaxial cylindrical conductor inside the conventional solenoid should be an appropriate configuration to generate the orthogonal magnetic field for tubular structures. When an orthogonal magnetic field excitation is adopted, different types of pick-up coils can be used to meet the requirements in different situations. For example, a self-differential mode pick-up coil can ensure similar sensitivity to various types of defects, a circumferential field pick-up coil can ensure enough high sensitivity to axial cracks, and a combination of them should be suitable for most occasions.

## Figures and Tables

**Table 1.** Simulation conditions.

**Model**	**Parameter**	**Value**
Tube	Outer diameter	82 mm
	Inner diameter	70 mm
	Wall thickness	6 mm
	Relative permeability	329.5
	Conductivity	0.5 × 10^7^ S/m

Sensor	Axial current density	8.2 × 10^6^ A/m^2^
	Circumferential current density	1.6 × 10^6^ A/m^2^
	Axial current waveform	Sinusoid, frequency 30 Hz and phase 0 °
	Circumferential current waveform	Sinusoid, frequency 30 Hz and phase 90 °

Defect	Direction	Axial and circumferential
	Size	Length 10 mm, width 0.5 mm and depth 1.8 mm
	Location	246 mm away from exciting coil

**Table 2.** Parameters of the test specimen.

**Type**	**Photography**	**Size**
Axial crack	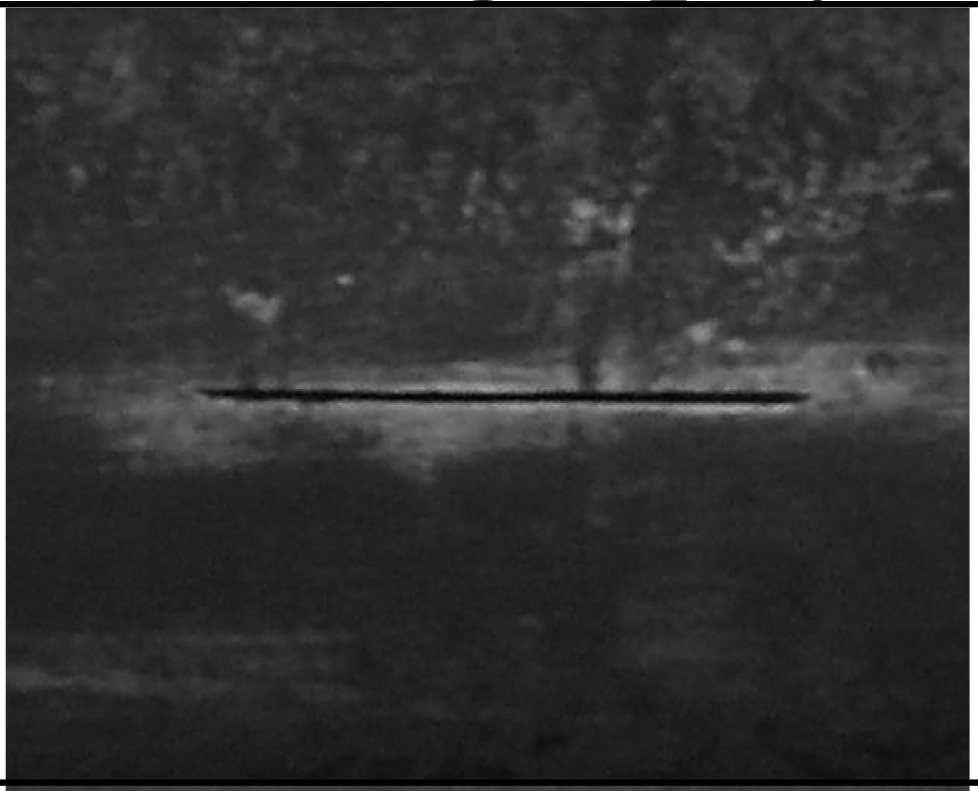	Length: 10 mm
		Width: 0.5 mm
		Depth: 15 %, 20 %, 40 % and 60 % of wall thickness
Circumferential crack	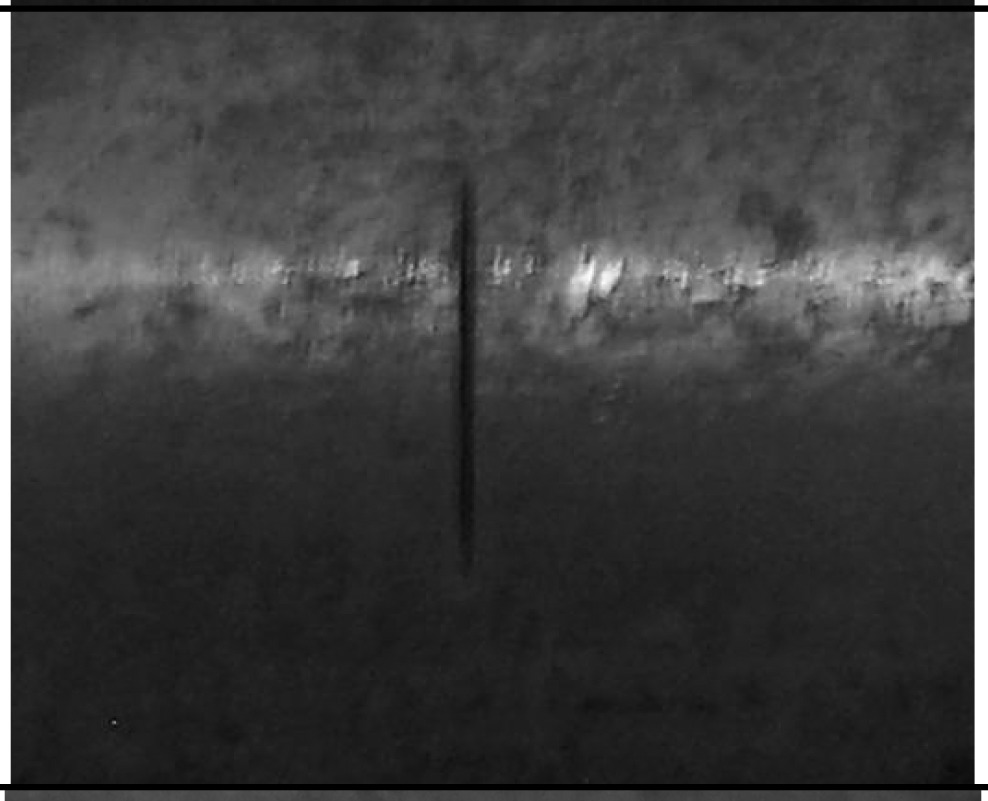	Length: 10 mm
		Width: 0.5 mm
		Depth: 15 %, 20 %, 40 % and 60 % of wall thickness
Circular defect	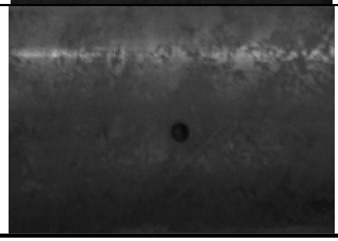	Diameter 3 mm
		Depth: 15 %, 20 %, 40 % and 60 % of wall thickness
